# PROTOCOL: Impact of summer programmes on the outcomes of disadvantaged or at risk young people: A systematic review

**DOI:** 10.1002/cl2.1343

**Published:** 2023-07-26

**Authors:** Daniel Muir, Becci Newton, Cristiana Orlando

**Affiliations:** ^1^ Institute for Employment Studies Brighton UK

## Abstract

This is the protocol for a Campbell systematic review. The objectives are as follows: based on the findings of the initial literature review regarding the types of summer programmes used to support disadvantaged or at risk young people, the outcomes they aim to affect and the goals of the funding organisations and research team, this review will seek to answer the following research questions: (1) to what extent does participation in summer employment programmes improve outcomes for disadvantaged or at risk young people, (2) to what extent does participation in summer educational programmes improve outcomes for disadvantaged or at risk young people, and (3) to what extent do the impacts of summer programmes vary based on the study, participant and intervention characteristics including the racial and ethnic make‐up of participants?

## 1 BACKGROUND

Many intervention studies of summer programmes examine their impact on the outcome domains of employment (e.g., Alam et al., 2013; Valentine et al., 2017) and education (e.g., Leos‐Urbel, 2014; Schwartz et al., 2020). But there is also growing interest in the impact of participating in summer programmes on the reduction of antisocial behaviour, including youth violence and criminal activity. Evaluating a Boston‐based summer employment programme using an randomised controlled trial (RCT), Modestino (2019) observed a reduction in violent‐crime and property‐crime arrests amongst programme participants—a pattern that persisted up to 17 months after participation. Furthermore, programme participants showed significantly increased community engagement, social skills, job readiness and future intentions to work (Modestino & Paulsen, 2019). This reduction in criminal behaviour was also observed in other summer employment programmes implemented in other cities across the United States (Davis & Heller, 2020; Heller, 2014; Heller, 2021). The initial evidence base on summer programmes offers some promise in terms of improving young people's outcomes and life chances. However, given the lack of a systematic review that estimates the extent of this impact, we cannot yet fully assert this. This current review seeks to fill this evidence gap.

In the body of available literature, there is no common definition of what constitutes a ‘summer programme’; most authors also do not propose a definition. There are though some common groupings of summer programmes. There are numerous ‘educational’ summer programmes—those that incorporate some form of academic instruction or support. Within this grouping there is significant heterogeneity in the type of intervention. Some educational programmes are focussed on remediation for students who are falling behind their peers, for instance New York's Summer Success Academy (see Mariano & Martorell, 2013). Others aim to support young people through transitions between stages of education—bridge programmes for incoming college students are particularly common in the US (e.g., Barnett et al., 2012), whilst outreach programmes commonly run by universities in the UK such as Aimhigher (see Horton & Hilton, 2020) look to raise the aspirations of school leavers and encourage entry to higher education. Other ‘employment’ summer programmes focus on transitions to the labour market, typically through some form of job placement often alongside wider employment support including careers guidance and wider skill development—see as examples the Boston Summer Youth Employment Programme (see Modestino, 2019; Modestino & Paulsen, 2019) or One Summer Chicago Plus (see Davis & Heller, 2020; Heller, 2014; Heller, 2021). Whilst there is wide variation in the features of different types of summer programmes, the literature documents some areas where there are commonalities within and across summer programme types, namely:

- the period in which the programme is delivered;
- the programme duration;
- the type of organisation delivering the programme;
- the programme participants;
- the types of tasks and activities included in the programme; and
- the programme's target outcomes (primarily short‐term).

For the purposes of this review, the researchers considered these features to construct operational definitions for different types of summer programmes.

### 1.1 Policy relevance

An initial literature review to scope existing evaluations of summer programmes identified that they may result in policy‐relevant outcome across the following domains:

- education (e.g., school participation, school completion, academic attainment, school readiness)
- employment (e.g., job readiness, soft skills, unemployment, job search skills)
- violence and offending (e.g., likelihood of reoffending, likelihood of involvement in illegal activity)
- socioemotional (e.g., resilience, confidence, social skills, community engagement, emotion management)
- health (e.g., understanding of health issues, such as substance abuse, physical activity, nutrition and condition management).

This review is anchored on the Outcomes Framework of the Youth Endowment Fund (YEF)—which focuses on reducing youth offending. It also takes account of the outcomes prioritised by Youth Futures Foundation (YFF)—which focus on supporting better employment for young people.

Given the early evidence showing reductions in criminal activity due to young people's participation in summer employment programmes (see Davis & Heller, 2020; Heller, 2014; Heller, 2021; Modestino, 2019; Modestino & Paulsen, 2019), a systematic review and meta‐analysis would be an appropriate next step to be able to verify the positive impact observed in previous studies but also to estimate the magnitude of this positive impact (if it is indeed found to be present). However, we propose to expand the coverage of this systematic review to also include summer educational programmes (e.g., summer school, summer learning programme), as well as to look at a broader set of outcomes across the policy‐relevant domains outlined above.

The inverse relationship between educational outcomes and youth violence has been extensively documented (Bushman et al., 2016). Given the currently mixed evidence regarding the effect of summer educational programmes and summer job programmes on educational outcomes (see Barnett et al., 2012; Kallison & Stader, 2012; Gonzalez Quiroz & Garza, 2018; Lynch et al., 2021; Sablan, 2014; Terzian et al., 2009), including educational summer programmes, this review presents an opportunity to examine their impact—though indirect—on youth violence. Education and employment are themselves linked and interact in deterring the production of antisocial behaviour (Lochner, 2004), a relationship acknowledged by the Outcomes Framework of both YEF and YFF—the expert reference group for the development of YEF's Outcomes Framework acknowledged that engagement in schooling is one of the most important factors in protecting young people from crime and violence. Those young people that end up being involved in the youth justice system are also disproportionately likely to have mental health problems including anxiety and depression, with there also being clear evidence of the links between work and health and socio‐emotional wellbeing (Waddell & Burton, 2006), and education and health and socio‐emotional wellbeing (Brooks, 2014; Department of Health, 2008). In light of this interrelatedness, it makes sense to consider both education‐oriented and employment‐oriented summer programmes in this systematic review, and to consider their effects across a wide range of highly interrelated outcome domains through direct, moderated and indirect effects. This is consistent with contemporary theories of youth development (Lerner & Castellino, 2002; YFF, 2021).

Disadvantaged young people are those who are at risk of poorer outcomes, including educational, economic, health and social outcomes, as a result of one or more adverse situational and behavioural factors faced in childhood and in the transition to adulthood. Situational factors that increase risks include race and ethnicity, low socio‐economic status, low parental attainment, being in care or a carer, and having disabilities or health conditions including mental health conditions. Behavioural factors that increase risk include involvement in crime or anti‐social behaviour, a low level or lack of parental support, truanting and being excluded from school, teenage pregnancy, and poor school performance in early years (Kritikos & Ching, 2005; Machin, 2006; Maguire & Newton, 2011, cited by Newton et al., 2010; Pring et al., 2009; Rathbone/Nuffield Foundation, 2008).

‘Educational’ and ‘employment’ summer programmes warrant considering together within this review as the contexts of and mechanisms employed by these programmes are often similar, and whilst there may be differences in the proximate outcomes they typically aim to achieve (educational programmes are typically focussed on educational attainment, and successful completion of and transitions between stages as their primary outcomes whilst employment programmes are typically focussed on entry to employment and labour market outcomes), as discussed these outcomes are highly interrelated with these programmes having a range of indirect effects on their participants. Additionally, any variation in the outcomes typically achieved by different summer programme types is important for policymakers to be aware of.

### 1.2 How the intervention might work

#### 1.2.1 Rationale for delivery and key assumptions

Summer programmes aim to improve the outcomes of young people through offering them alternative provision; that is, additional to the usual curriculum for their age and stage (which may be considered ‘service as usual’) (Barnett et al., 2012; EEF, n.d.; Heller, 2021; Hutchinson et al., 2001; Modestino, 2019; Tarling & Adams, 2012). The intention is to avoid interference with the standard curriculum and to build additional support to improve outcomes in ‘service as usual’ including progression through education as well as into the labour market. An assumption is that targeted young people will find programmes attractive and engage in them, with a further assumption that they will be supported by their families and/or carers to do so.

While the characteristics of the target group may vary—from those with offending histories or at risk of these (Modestino, 2019; Tarling & Adams, 2012), to those with low attendance and low attainment (Hutchinson et al., 2001) and to what is sometimes described as the grey or middle group who fail to grab attention but also are at risk due to not having firm ambitions (Barnett et al., 2012)—there is also recognition that the selected target group is not engaging with service as usual as effectively as other groups, or they may not be engaging at all. Therefore, the assumption is that an alternative approach is required to foster more positive engagement or re‐engagement to achieve outcomes.

Summer education programmes may focus on ‘catch up’ with aims of closing the attainment gap for disadvantaged learners (EEF, n.d.; Tarling & Adams, 2012), or be aimed to support transitions between education phases (Hutchinson et al., 2001) and to accelerate achievement in the next education phase (Barnett et al., 2012). They may offer learning in an alternative format (Tarling & Adams, 2012), as part of smaller groups or with more staff support which can lead towards better attainment (EEF, n.d.). The underlying assumption, as identified by the Education Endowment Foundation (EEF) toolkit (n.d.), is simply that more time in school/education leads to better educational outcomes.

Summer job programmes may share similar aims. For example, Alam et al. (2013) explores summer job programmes that aim to support and improve transitions to the next stage of education. The assumption is that the job placement creates an early insight into the labour market that builds ambition. This in turn increases understanding of the importance of educational credentials to good quality work. As a result, motivation for achieving in the next phase of education is increased. Summer job programmes may also aim to divert or distract those who have been involved in or are at risk of offending away from harmful or unproductive activities (Leos‐Urbel, 2014; Modestino, 2019). The underlying assumption is that through providing alternative uses for the time over summer that otherwise would be unallocated reduces the risk of that time being used for criminal activity.

#### 1.2.2 Mechanisms

There are a number of mechanisms through which summer programmes work, with many of these shared across job and education programmes. This stems in large part from common intermediate outcomes relating to personal and social development, and vocational and applied skill acquisition. For example, Modestino (2019) identifies a mechanism through building aspiration, self‐belief, emotion control and a longer‐term work ambition. The summer job encourages young people to improve their engagement with education as a precursor to achieving newly found higher quality employment goals. This in turn leads to better attainment—which was an outcome not originally anticipated. The commonalities with the summer education programme concern the soft skill development including self‐esteem and confidence, emotion control, leadership skills, communication, problem‐solving, and responsibility and time management (Hutchinson et al., 2001; Leos‐Urbel, 2014).

A common mechanism in the summer programmes targeted at disadvantaged or at risk young people concerns the opportunity to form better relationships. In summer education programmes, this can result from the group of young people formed for the programme (EEF, n.d.; Hutchinson et al., 2001). It also results however where delivery teams are new to the young people. Hence, in summer education programmes that are delivered by staff who are different from those in service as usual, there is a chance to re‐set engagement with adults, which can then set the tone for the next stage of service as usual. In summer job programmes, the adult relationship is formed with employees in the employing organisation. This, along with the employers’ expectation of performance from the young person, builds responsibility, maturity and self‐esteem (Alam et al., 2013; Modestino, 2019). Improved interpersonal relationships might also contribute towards feeling more settled thereby supporting improved wellbeing—although evidence for these outcomes is weak (Terzian et al., 2009). In both summer job and summer education programmes, financial incentives can be a mechanism for change (Barnett et al., 2012; Modestino, 2019). Providing financial recognition can have an important effect on how the opportunity is valued within the young person's household—which can support engagement from families and/or carers, as well as providing a reward for the young person's time. Financial incentives may also help to alleviate financial constraints on future education, increasing investment in human capital and improving longer‐term outcomes.

Location is an important mechanism to the outcomes for some summer programmes. For summer job programmes, young people are exposed to the world of work, and are located in an organisation for a job placement. This builds familiarity and confidence in this new setting as well as increases expectations for conduct in this adult environment (Heller, 2021; Modestino, 2019). Where summer education programmes support transitions to the next phase of education they may take place on the campus of that next phase. This similarly builds familiarity and confidence to be in this new environment. In these programmes, building familiarity with the campus and the services available can increase likelihood to seek out and use support services, which in turn provides crucial underpinning to sustaining this destination, that is, reducing the likelihood of drop‐out, particularly important when transitioning to higher education. Finally, summer education programmes may be located in alternative settings, such as the outdoors, providing a different context for learning that can support young people to engage differently and to achieve in this environment, thereby building confidence for learning in the traditional classroom setting (Tarling & Adams, 2012; Terzian et al., 2009).

These are all positive causal mechanisms to the achievement of outcomes, however some studies identify the potential for negative effects from summer programme participation resulting from some of these mechanisms. This includes, for example, Alam et al. (2013) who suggests that requiring disadvantaged young people to attend summer job programmes at a time when their peers are at rest and on vacation can leave them exhausted and therefore not well placed for the start of the new term. This is a risk that intuitively reads across to summer education programmes. Consequently, duration and intensity of the programmes will be important contextual factors in the analysis. Alam et al. (2013) also indicates that the positive effect on attainment established by Modestino (2019) may not result from all summer job programmes. Rather than build motivation through understanding why education is important, the ability to earn ‘easy’ money from summer jobs may deter young people from engaging in their further studies. Quality of and safeguarding in the job placement are also a key consideration to ensure young people do not see negative consequences from encountering poor social behaviour among standard employees. For economically disadvantaged young people a further consequence of being part of summer job or education programmes may be that they are unavailable for activities such as standard employment that is better paid. This may have consequences for short‐ and long‐term financial returns as well as for engagement and attrition in programmes.

#### 1.2.3 Outcomes

Considering the mechanisms through which summer programmes may affect positive outcomes over the longer‐term, summer job programmes provide meaningful employment experiences which can provide alternative pathways for disadvantaged young people, opening up economic opportunities to them which, because of their disadvantage, may be limited outside of public interventions relative to more advantaged youth (Modestino & Paulsen, 2019). Summer education programmes, through the mechanisms discussed above, may lead to improved academic attainment in following phases of education, which will also improve future economic opportunities by increasing the individuals’ skills and desirability in the labour market. As a result, both summer education and job programmes can improve violence and offending outcomes—in improving the individuals’ economic opportunities and this expectations regarding their future quality of life, they become less likely to offend as the opportunity costs of the punishment that may result are now greater (Heller, 2014). Improved economic opportunities resulting from participation in a summer programme may also affect positive health and socio‐emotional outcomes, by potentially improving nutritional choices, reducing anxiety and stress, and increasing self‐confidence and one's sense of self‐worth as a result of increased financial resources. Given the interrelatedness between education, employment, violence and offending, health and socio‐emotional outcomes, intermediate improvements in outcomes within one domain as a direct result of participation in a summer education or employment programme is likely to result in improved outcomes across the other domains.

## 2 OBJECTIVES

### 2.1 Research questions

Based on the findings of the initial literature review regarding the types of summer programmes, the outcomes they aim to affect, and the goals of the funding organisations and research team, this review will seek to answer the following research questions:

*Meta‐analysis*

1. To what extent does participation in summer employment programmes:
  a. improve violence and offending outcomes?
  b. improve educational outcomes?
  c. improve employment outcomes?
  d. improve socioemotional outcomes?
  e. improve health outcomes?
2. To what extent does participation in summer educational programmes:
  a. improve violence and offending outcomes?
  b. improve educational outcomes?
  c. improve employment outcomes?
  d. improve socioemotional outcomes?
  e. improve health outcomes?
3. To what extent do the outcomes of summer programmes vary based on the study, participant and intervention characteristics including the racial and ethnic make‐up of participants?

*Thematic synthesis*

4. What are common features of summer programmes (employment and educational) that successfully produce positive outcomes? Which features contribute most to the achievement of:
  a. improve violence and offending outcomes?
  b. improve educational outcomes?
  c. improve employment outcomes?
  d. improve socioemotional outcomes?
  e. improve health outcomes?
5. In which contexts are summer programmes (employment and educational) most or least able to produce positive outcomes? Are any contexts more or less able to produce:
  a. improve violence and offending outcomes?
  b. improve educational outcomes?
  c. improve employment outcomes?
  d. improve socioemotional outcomes?
  e. improve health outcomes?
6. Which mechanisms inhibit or enable the effectiveness of summer programmes (employment and educational)? Are any mechanisms particularly important to achieving:
  a. improve violence and offending outcomes?
  b. improve educational outcomes?
  c. improve employment outcomes?
  d. improve socioemotional outcomes?
  e. improve health outcomes?
7. In what ways do targeting, retention, and dropout affect the attainment of outcomes of interest by summer programmes (employment and educational)?

### 2.2 Why is this review needed in light of existing reviews?

Whilst still limited, the evidence base regarding the impact of summer programmes is growing. There have been a number of intervention studies that examine the effect of summer employment programmes on antisocial behaviour amongst the youth (e.g., Davis & Heller, 2020; Heller, 2014, 2021; Modestino, 2019; Modestino & Paulsen, 2019). Findings of these individual intervention studies have been promising, showing a relationship between participation in summer employment programmes and reduced antisocial behaviour. However, the lack of a systematic review makes it difficult to ascertain this relationship and to estimate the extent to which positive behavioural outcomes can be attributed to participation in the programmes. This review offers an opportunity to also examine the impact of summer employment programmes on other outcomes that influence young people's life chances, such as education and employment – both outcome domains that at least some of the evidence on summer job programmes has examined – as well as violence and offending, socioemotional and health.

There is a well‐documented link between educational outcomes and youth violence (Bushman et al., 2016), so it is also important to take stock of summer programmes that seek to improve the outcomes of interest to this review. EEF performed a systematic review of summer schools and their impact on educational outcomes amongst 3–18 year‐olds for their Teaching and Learning Toolkit (EEF, n.d.), which found that summer schools had a moderate impact on educational outcomes. Other evidence of the positive impact of summer schools has also been conclusive (see Cooper et al., 2000; Lauer et al., 2006). However, evaluations of other forms of education‐oriented summer programmes have yielded more mixed results (see Barnett et al., 2012; Gonzalez Quiroz & Garza, 2018; Kallison & Stader, 2012; Lynch et al., 2021; Terzian et al., 2009). The inclusion of literature regarding education‐oriented summer programmes offers an opportunity to clarify the currently mixed evidence regarding their impact. There is also a need to update the existing evidence in light of policy changes, such as the transition to and implementation of the Raised Participation Age (RPA) policy in England in 2012—of the 59 studies included in the EEF systematic review, only five were published since 2012, with the most recent being published in 2014. Additionally, as previously detailed, summer education programmes may affect outcomes across domains other than education, which warrants an investigation of the impacts of summer schools and summer education programmes more broadly across a wider range of outcome domains. Furthermore, given the age range employed by EEF, summer schools, which constitute a large component of summer education programmes, have not been synthesised for their effects beyond the age of 18 and so current analyses do not cover any studies focused on post‐18 study which may be compensatory (catching up on what should have been achieved in compulsory schooling) or at the further or higher level.

RAND have also performed a systematic review of summer programmes, education and employment focussed as well as wellbeing and enrichment (McCombs et al., 2019), to support the Wallace Foundation's Summer Learning Toolkit (Wallace Foundation, n.d.). The review covered only interventions from the USA, and across age groups from pre‐kindergarten through to the summer before grade 12 (ages 17–18). Outcomes relating to academic achievement, academic and career attainment, engagement with schooling, social and emotional competencies, physical and mental health, and the avoidance of risky behaviour were considered, although no meta‐analyses were conducted. TASO also have a stream relating to summer schools for their Evidence toolkit, based on a collection of UK interventions focussed on transitions to higher education (TASO, n.d.), which finds that summer schools have a small positive impact on student aspirations and attitudes. The strength of evidence is emerging, that is, relatively weak, with many of the studies covered not employing robust experimental/quasi‐experimental designs.

Considering the wider content of summer programmes, which can be judged to focus on ‘enrichment’, Malhotra et al. (2021) conducted a robust systematic review of sports interventions programmes and their impacts on outcomes such as offending, anti‐social behaviour or violence. Given that they include interventions whose main component is participation in sports or physical activities, these interventions should not fall within the scope of this review. Other forms of summer enrichment programme—if found to be distinctive from education or employment—will be considered against the definitional criteria for this review including capture of relevant outcomes. Researchers will judge closest proximity to education or employment programmes for any that meet the inclusion criteria.

## 3 METHODS

### 3.1 Overview of approach

We intend to lead a systematic review with meta‐analysis. Four key stages will underpin this:

1. search the appropriate literature through an agreed list of search terms;
2. select relevant studies based on specified and agreed inclusion and exclusion criteria;
3. extract relevant evidence using an agreed protocol;
4. synthesise and interpret the evidence to inform high quality, user friendly, accessible, engaging, relevant and useful reviews.

This systematic review will examine out‐of‐school‐time programmes conducted throughout or at some point during the summer months (by which we mean the period in which the long vacation takes place between academic years or after the final academic year before moving into economic activity). These programmes include summer employment programmes and summer educational programmes.

We are interested in how these programmes improve outcomes amongst disadvantaged and at risk youths as these young people are at risk of poorer outcomes in later life, including educational, economic, health, and social outcomes, as a result of one or more adverse situational and behavioural factors faced in childhood or as a young adult. While the experience of even one disadvantage factor may lead to young people facing difficulties in transitioning into adulthood, disadvantage factors often interact and compound each other leading to severe adverse impacts for young people and society, including decreased productivity and the perpetuation of poverty and social exclusion. Disadvantaged young people are also twice as likely to be long‐term NEET as their better off peers (Gadsby, 2019).

As is standard with systematic reviews, we will consult content experts to refine search terms and locate additional databases to search. For this study, content experts will come from this review's advisory group.

To determine whether summer programmes produce improvements in outcomes of interest, and to estimate the magnitude of this relationship (where it exists), we will conduct a meta‐analysis, employing the random effects model (see research questions #1 through #3).

Since this systematic review also seeks to identify components and features shared across successful summer programmes (see research questions #4 through #7), we will also attend to qualitative evaluations of the interventions examined in other studies that meet the inclusion criteria for meta‐analysis, expanded by examples found in the UK where these meet the inclusion criteria except for study design and there is no evaluation of the intervention that does meet the full list of inclusion criteria. This approach, recommended by the expert panel, seeks to ensure the review can tap into the UK context, particularly for implementation data. Where outcomes of interest are not observed or covered by these studies, they will not feed into the analysis of the causal pathway.

### 3.2 Search strategy

IES will search various electronic databases to identify studies for inclusion in the review. We will search Scopus, PsycInfo, Child Development and Adolescent Studies (CDAS), the Education Resources Information Center (ERIC), and the British Education Index (BEI). We will explore wider resources including the current unpublished updated YFF EGM, which includes studies from the 3ie Evidence and Gap Map/Kluve synthesis, the summer school streams of EEF's Teaching and Learning Toolkit and TASO's Evidence toolkit, and RAND's summer programs evidence review (McCombs et al., 2019) that supports the Wallace Foundation's Summer Learning Toolkit. We will search the Pathways to Work Evidence Clearinghouse, Clearinghouse for Labor Evaluation and Research; Office of Planning, Research and Evaluation, Administration for Children and Families; MDRC; the National Bureau of Economic Research (NBER); and the Trip database. We will search the most relevant journals—the *Journal of Youth Studies*, *Youth & Society*, *International Journal of Adolescence and Youth*, *Journal of Social Policy, and Youth*.

To locate grey literature, we will search Google Scholar (manually title appraise the first 100 publications identified) alongside selected sites-gov.uk, gov.scot, wales.gov.uk, northernireland.gov.uk, gov.ie, National Lottery Community Fund, Care Leavers Association, Children's and Young People's Centre for Justice, JRF, Centre on the Dynamics of Ethnicity, Nuffield Foundation, RSA, Centrepoint, Youth Employment UK, Impetus, Edge, Education and Employers, National Foundation for Educational Research (NFER), the Sutton Trust, and TASO. For each of these latter sources, we will manually title appraise the first 50 examples for relevance.

Several other databases that might be expected to be included in this list are not as during piloting and testing of the search string, these additional databases surfaced limited/no additional studies of relevance. These additional databases cover Medline, the World Health Organization's International Clinical Trials Registry Platform, databases in the US National Library of Medicine including ClinicalTrials.gov, Social Care Online from SCIE, Epistemonikos, the libraries of Cochrane and the Campbell Collaboration, additional What Works Centres including the What Works Wellbeing, the Wales Centre for Public Policy and the What Works Centre for Children's Social Care. Scopus is available through Elsevier, PsycInfo is available through the American Psychological Association, CDAS and BEI are available through EBSCO, the Journal of Youth Studies and the International Journal of Adolescence and Youth are available through Taylor & Francis Online, and the Journal of Social Policy is available through Cambridge Uni Press. In implementing our search strategy, we will follow the template and guidance provided by the Campbell Collaboration (Kugley et al., 2017).

We will use the following basic string to interrogate the identified databases:

(“summer school*” OR “summer learn*” OR “summer education*” OR “educational summer” OR “summer bridge” OR “summer employ*” OR “summer work” OR “summer place*” OR “summer job*” OR “summer apprentice*” OR “summer intern*” OR “summer camp*” OR “summer program*”) AND (“youth” OR “young” OR “child*” OR “student*” OR “pupil*” OR “teenage*” OR “adolescen*” OR “juvenile”) AND (“disadvantage*” OR “vulnerab*” OR “at risk” OR “at‐risk” OR “marginalised” OR “marginalized” OR “youth offend*” OR “young offend*” OR “delinquent” OR “anti‐social”)

This was developed through initial piloting of the string and discussions with the review's advisory group. We will use the full‐search string where possible. Where databases limit the length of the search string that can be used (either through physical limits or where the search function is too sensitive so that inputting the full search string is inappropriate), we will employ a hierarchical approach, inputting as many of the key terms (ordered in terms of relevance) as possible, starting with those relating to the intervention before adding those relating to the population of interest (first age‐related, second disadvantage‐related). Depending on the size of the database and its subject‐matter focus, should a full advanced search not be possible we will search for just ‘*summer*’ or run individual searches for each of the terms relating to the programmes of interest, that is, ‘*summer school*’ then ‘*summer learn*’, and so forth, through to ‘*summer program*’—the former approach would be followed for the Care Leavers Association whilst the latter would be used for gov.uk for instance. We will search in all fields of each record within each database unless in certain cases the number of hits is excessive and the relevancy of hits is too low—in these instances, we will search within the abstract, title and/or key words.

The terms relating to the intervention type and the age/demographic group of participants are the predominant terms used in the literature. We have also piloted a series of terms relating to the disadvantage characteristics for our population of interest to test whether these capture all of the literature of interest – some studies for instance may only use specific disadvantage terms such as ‘*poverty*’ or ‘*ethnic minority*’ or ‘*special educational needs*’, therefore risk not getting picked up by the search string. In each of the databases where it is possible to input the full search string, we tested using 40 different search terms relating to specific forms of disadvantage. These additional searches yielded 1229 additional hits versus the original shorter search string—of these, only six of these were studies that merited full text screening.

Where databases permit, we will apply date limiters to only include studies published since 1 January 2012, that is, approximately the last decade's worth of research and covering the transition to and implementation of the Raised Participation Age (RPA) policy in England which affects education and training participation, and up to 31 December 2022. This will maximise the policy relevance of this review's findings. We will include English language studies only—this is common practice across systematic reviews (Jackson & Kuriyama, 2019) despite potentially introducing bias to the review, although it has been shown that excluding non‐English language studies does not affect the main findings from meta‐analyses (Morrison et al., 2012). Additionally, the focus of the review on high income countries should also alleviate this as an issue, as studies based on interventions in high income countries may be more likely to be available in English, either primarily or as an alternative to the main non‐English language version. Furthermore, the saturation principle (discussed further in relation to the study design inclusion criteria) provides further support for this.

Searching Scopus will surface relevant conference proceedings. Dissertations will be included in the review should they be surfaced through the process detailed above, but we will not search dissertation‐specific databases such as ProQuest due to the volume of and low degree of relevance of the literature collected in this database. We will search the references of the most relevant evidence reviews, namely the EEF and TASO toolkits and RAND's summer programs evidence review (McCombs et al., 2019) supporting the Wallace Foundation's toolkit, as well as any additional systematic reviews surfaced through the search process.

The specific implementation of the search strategy across each of the databases searched will be documented transparently in the technical report. As examples of the specific search strategy, in Scopus we will use the following string:

TITLE‐ABS‐KEY ((“summer school*” OR “summer learn*” OR “summer education*” OR “educational summer” OR “summer bridge” OR “summer employ*” OR “summer work” OR “summer place*” OR “summer job*’ OR “summer apprentice*” OR “summer intern*” OR “summer camp*” OR “summer program*”) AND (“youth” OR “young” OR “child*” OR “student*” OR “pupil*” OR “teenage*” OR “adolescen*” OR “juvenile”) AND (“disadvantage*” OR “vulnerab*” OR “at risk” OR “at‐risk” OR “marginalised” OR “marginalized” OR “youth offend*” OR “young offend*” OR “delinquent” OR “anti‐social”)) AND (LIMIT‐TO (PUBYEAR, 2022) OR LIMIT‐TO (PUBYEAR, 2021) OR LIMIT‐TO (PUBYEAR, 2020) OR LIMIT‐TO (PUBYEAR, 2019) OR LIMIT‐TO (PUBYEAR, 2018) OR LIMIT‐TO (PUBYEAR, 2017) OR LIMIT‐TO (PUBYEAR, 2016) OR LIMIT‐TO (PUBYEAR, 2015) OR LIMIT‐TO (PUBYEAR, 2014) OR LIMIT‐TO (PUBYEAR, 2013) OR LIMIT‐TO (PUBYEAR, 2012)) View less

That is, the full search string with search limits on publication date, whilst in Google scholar due to the 256 character limit on search string we will use the following:

“summer school*” OR “summer education*” OR “educational summer” OR “summer bridge” OR “summer employ*” OR “summer work” OR “summer place*” OR “summer job*” OR “summer intern*” OR “summer camp*” OR “summer program*”

That is, with just the terms relating to the intervention excluding ‘summer apprentice*’ and ‘summer learn*’ individually produce the least hits and whose removal does not reduce the total number of hits, and with limits on the publication date selected manually after the search is performed.

We will consult with subject matter experts (these come from the study's advisory group made up of experts from the Campbell Collaboration, YEF and YFF) before finalising these search terms although we have undertaken initial piloting ahead of developing this protocol.

We will use pearling to establish whether process studies are available for those studies selected for the review, as well as including process studies related to UK interventions that are eligible for inclusion other than on study design. This will mean they are not included in the meta‐analysis. Recommendations from experts will be accepted alongside those generated through the search process.

### 3.3 Selection criteria

#### 3.3.1 Population

Interventions should have been conducted with individuals aged 10–25 years old (based on whether students typically are/turn this age in the academic year they are in where necessary). The upper end of the age range covers the ages at which the vast majority of individuals in the UK will have exited education and entered economic activity as well as corresponding to the upper end of the age range of interest to YFF, whilst the lower end covers the transition to secondary education in the UK as well as the lower end of the age range of interest to YEF.

The young people taking part should also be considered disadvantaged or at risk. These terms are used widely throughout the literature despite not being strictly defined. We will not limit ourselves with a concrete definition of disadvantage or at risk characteristics. Rather, we will allow the literature to dictate which groups fall under this criterion. We will consider all groups who face disadvantage or are at risk of poorer outcomes across the domains of interest compared to the wider population, which may include but not be limited to racial and ethnic minorities, individuals of low socio‐economic status, individuals that have experienced care, students with Special Educational Needs, individuals with health conditions or disabilities, as well as those that have already offended or have experience of the criminal justice system, and those who are already experiencing poorer outcomes including poor academic performance or those truanting or being excluded from school.

#### 3.3.2 Intervention

As previously noted, we are interested in two main types of summer programme surfaced by the initial literature review conducted—summer employment and summer educational programmes. For the purposes of this review, these programme types are operationally defined as follows:

- **Summer employment programme:** an out‐of‐school‐time programme that takes place during the summer months in whole or in part and includes a fixed‐term job placement;
- **Summer educational programme:** an out‐of‐school‐time programme that takes place during the summer months in whole or in part, where content is majority administered through education‐focused instruction.

By summer months we are referring to the period in which the long vacation takes place between academic years or after the final academic year before moving into economic activity—interventions that take place during the summer and are targeted at individuals that have already transitioned into the labour market are not of interest. Summer programmes that are a part of a wider intervention, for instance including term‐time provision, are eligible for inclusion although the features, mechanisms and/or outcomes of the summer programme should be able to be separated out from the other components of the intervention, and/or the summer programme should constitute a substantial enough component of the whole intervention for it to be reasonable to be included in the review. This will be determined on a case‐by‐case basis ‐ the reasoning behind decisions for any marginal cases will be made transparent.

Sports programmes (which according to a broader definition could be considered educational programmes) that were subjected to the systematic review of Malhotra et al. (2021) will not be included—given their definition of sports programmes though these should not overlap with the interventions of interest to us—however, interventions that meet the definition of summer employment or summer education programmes which also feature sports activities will be included. Education‐focused instruction that does not serve some academic purpose, for instance cycle training programmes, will not be considered—programmes where the education‐focused instruction relates to understanding of/familiarisation with transition to, for example, higher education, are eligible as they employ various mechanisms of interest and in the broader sense constitute a summer education and not enrichment programme. Residential programmes which aim to achieve this through familiarising students with a new environment will be considered provided that there is some form of guided instruction and the programme is not solely focussed on enrichment activities. Programmes such as reading challenges or book gifting programmes without guided instruction will also not be considered.

These definitions should make the programme types mutually exclusive. When it is unclear which category an intervention falls into, we will allow the literature to define the programme type, that is, if an intervention describes itself as an educational programme then we will treat it as such, or we will judge closest proximity to the education or employment programme definitions for any interventions that meet the inclusion criteria that do not identify themselves as one or the other. We will include studies that evaluate a summer employment or summer educational programme.

Interventions for inclusion should be targeted at the population groups identified above. Universal interventions where disadvantaged or at risk young people fall into the intervention population but are not specifically targeted will not be considered. The interventions should also be provided directly to the population of interest, as opposed to indirectly through a third party such as their parents or teachers.

#### 3.3.3 Comparison group

Primary studies will be included in the systematic review where they draw on a comparison group (QED) or control group (RCT). The comparison group will be young people who do not participate in summer programmes (provided as part of evaluation, they may participate in a summer programme outside of the evaluation) but who are similar to those who do participate. It is expected that primary studies will typically draw comparison with groups experiencing business as usual (BAU). Being able to access comparative analysis between intervention strands within primary evaluation reports will be crucial for studies to be included in the review. This requirement will be dropped for studies evaluating UK‐based interventions which meet all the criteria for inclusion except for study design.

#### 3.3.4 Outcomes

The systematic review will examine the impact of different types of summer programmes across the five outcome domains of interest previously discussed: (1) violence and offending; (2) education; (3) employment; (4) socioemotional; and (5) health (where these are included alongside other outcomes of interest). To be included in this review, a study must evaluate the intervention according to an outcome within at least one of these domains, with those studies considering health outcomes included only where outcomes within another domain are also covered. This is to avoid ‘weightloss camps’ or programmes which are aimed solely at helping young people to manage health conditions/disabilities—if these health interventions also only look to affect socio‐emotional outcomes which can be thought of as direct consequences of potential health outcomes as opposed to distinctly separate outcomes (for instance, weightloss camps may also consider impacts on confidence and self‐esteem), then these will also not be considered. Within the context of this systematic review, violence and offending also includes anti‐social behaviour.

The initial literature review identified that the outcomes measured as part of the evaluation of relevant interventions were mostly relatively short term, with studies often not following‐up after programme end. As such, outcomes that would usually be considered as intermediate, such as the acquisition of skills and attributes outlined in YEF's Outcomes Framework, will also be considered as outcomes of interest to this review.

The specific outcomes that are of interest will be guided by the Outcomes Framework of YEF and the outcomes of interest to YFF, as well as the initial literature review to scope existing evaluations of summer programmes. These include:

- Violence and offending—reduced offending and reoffending; reduced likelihood of carrying weapons. Note that both the severity and intensity of violent and offending behaviour will be appropriately considered, as will the differentiation between self‐reported measures and measures based on recording from the police and/or criminal justice system. When considering impacts on the rate of violent or offending behaviour, we will group types of violent and/or offending behaviour appropriately based on their form and severity.
- Educational—education & qualification completion (including performance and attainment in courses/exams); access to/in education (including application, participation, and completion in courses); education quality; technical skills & vocational training; improved study skills and academic mindset; improved critical and analytical skills.
- Employment—employment status; whether actively seeking employment; employment expectation; whether found appropriate employment; hours worked; job quality; earnings & salary; development of work appropriate ‘soft‐skills’ including job‐search skills.
- Socioemotional—resilience and persistence; increased confidence; improved behavioural adjustment indicators; improved social skills; community engagement; ability to manage emotions and resolve conflicts.
- Health—better understanding of health issues including substance use, physical activity, and nutrition; improved family well‐being; improved access to health‐related support services.

Where relevant, outcomes from longitudinal analyses will be differentiated from those from correlational or cross‐sectional analyses, more explicitly so as part of the thematic synthesis as these outcomes are more naturally differentiated in the meta‐analysis.

#### 3.3.5 Study design

Experimental (RCT) and quasi‐experimental designs (QEDs) (including but not limited to RDDs, DIDs and matching approaches) as part of evaluation studies with a robust and credible comparison group will be included. Empirical studies looking at the implementation of the approach or process evaluations will be included in the review to examine implementation questions—these will be sourced from the pearling of included counterfactual impact evaluations. Qualitative evaluations of this latter type of UK‐based interventions where these meet the inclusion criteria except for study design will also be included.

To include qualitative evaluations in the thematic synthesis where we will consider how the contexts of summer programmes affect the outcomes achieved through various mechanisms, we require a credible indication of what impacts the intervention achieved. Therefore, we require qualitative studies to be linked to a robust impact evaluation. As this review is most interested in policies in the UK where there has until more recently, with the development of the What Works movement, been a lack of tradition of robust impact evaluation, this requirement is suspended in this context. Additionally, initial piloting of the search string to support the development of this protocol suggested that applying requirements these requirements on study design would still result in a substantial amount of literature to review. Theoretical saturation, a well‐established approach in qualitative primary research (e.g., Hennik & Kaiser, 2022; Morgan et al., 2002), suggests that beyond a certain point any additional qualitative evaluations would not provide new information to inform the findings of the review. As such, not including qualitative evaluations of eligible interventions that do not fulfil the requirements on study design is unlikely to affect the main findings of the thematic synthesis, and any impact is outweighed by the certainty with which we are able to make assertions relating to outcomes given that the qualitative and process information is related to an intervention that has been subject to a robust impact evaluation.

#### 3.3.6 Setting

The systematic review will cover summer programmes implemented in high‐income countries (as defined by the World Bank for July 2022 to July 2023) at any level (i.e., national, regional, and local programmes).

The PICOSS inclusion and exclusion criteria detailed above are summarised in Table 1.

**Table 1:** Summary of PICOSS criteria.

Title 1	Inclusion criteria	Exclusion criteria
Population	Young disadvantaged or at risk people aged between 10 and 25.	Young people aged less than 10 or more than 25 or not disadvantaged or at risk.
Intervention	**Summer employment programme**: an out‐of‐school‐time programme that takes place during the summer months in whole or in part and includes a fixed‐term job placement.	Programmes that do not fulfil the criteria of either a summer employment or summer educational programme.
	**Summer educational programme**: an out‐of‐school‐time programme that takes place during the summer months in whole or in part, where content is majority administered through education‐focused instruction.	
Comparison	Treatment as usual, another intervention, no intervention, or wait‐list control.	Studies that cover a population that is different in observable characteristics and that receive an alternative intervention not tracked by evaluation. Studies that mobilise non‐counterfactual measures except eligible studies of UK‐based interventions.
Outcome	Studies that examine: (1) violence and offending; (2) academic; (3) employment; (4) socioemotional; or (5) health outcomes.	Studies that examine other outcomes while not covering the outcome domains of interest. Studies that only consider health outcomes or health outcomes plus socio‐emotional outcomes that are direct consequences of health outcomes.
Study design	Randomised controlled trials (RCTs) including individual and cluster level randomisation. Step‐Wedge designs with random time allocation. Non‐equivalent control group designs using parallel cohorts that adjust for baseline equivalence Difference‐in‐Difference estimation Interrupted time‐series Synthetic control group methods Studies based on: ‐ covariate matching; ‐ propensity score‐based methods; ‐ doubly robust methods^a^; ‐ regression adjustment; ‐ regression discontinuity designs; and ‐ instrumental variable estimation. Qualitative studies and economic evaluations will be included if they are conducted as part of a qualifying study and will be used only to generate hypotheses, inform us about the interventions and populations, and inform or deepen our understanding of the quantitative findings. They will be included however if they are evaluating UK‐based interventions and are identified via the searches or recommended to this study by experts.	Non‐primary studies (except studies of this type that are evaluating UK‐based interventions), including: Literature reviews; Systematic reviews; Meta‐analysis; and Non‐primary QEDs. Studies without a valid counterfactual, including designs that do not include a parallel cohort that establish or adjust for baseline equivalence (except studies of this type that are evaluating UK‐based interventions), including: Single group pre‐post designs; Control group designs without matching in time and establishing baseline equivalence; Cross‐sectional designs; Non‐controlled observational (cohort) designs; Case‐control designs; Case studies/series; and Surveys.
Setting	Studies that are undertaken in high income countries, as defined by the World Bank.	Studies that are not undertaken in high income countries, as defined by the World Bank.
Other	Studies that are published in English.	Studies that are not published in English.
	Studies published since 2012 up to the end of 2022.	Studies published before 2012 or since 2023.
	Published studies.	Unpublished studies.

^a^ “Combines a form of outcome regression with a model for the exposure (i.e., the propensity score) to estimate the causal effect of an exposure on an outcome” (Funk et al., 2011, p. 761).

### 3.4 Study selection process

Once the longlist has been compiled in Covidence, we will screen the abstracts and summaries against the agreed inclusion/exclusion/quality/scope/applicability criteria. Titles and abstracts will be initially screened by two reviewers. Where a conflict arises, it will be resolved by a third reviewer. Where evidence fails to meet criteria, it will fall out‐of‐scope. The output will be a sub‐set of the search database tagged ‘for review’. Reasons for exclusion will be noted.

Subsequently, the full text of all potentially eligible evaluations will be retrieved and reviewed for eligibility, independently by two members of the team using our a priori eligibility criteria. Full‐text review will be completed with one reviewer for inclusion and two reviewers required for exclusion, as per Cochrane rapid review methods.

### 3.5 Study quality assessment

We will lead a critical appraisal of each study (impact and process evaluations) which will be conducted independently by a pair of reviewers who will follow the same procedure used at screening and coding phases to reconcile disagreements (discussion and help of a tie breaker).

As recommended by the EGM protocol, the Quality Assessment of Impact Evaluations Tool (Saran et al., 2020) will be used to evaluate impact evaluation studies. The checklist contains seven items to be rated as: high confidence, medium confidence or low confidence. Following the approach in the EGM, four items—*study design* (related to confounders); level of *attrition* or losses to follow up; *definition of outcomes*; and *baseline balance reports*, will be prioritised in decision making, although the other three items—adequate *sample size*; *definition of intervention*; and *overall confidence*, will still be considered.

Similarly, we will use the ‘Questions for Process Evaluations’ as set out in the EGM protocol to assess the qualitative and process evaluation studies. These cover whether methodology is described and appropriate to the research questions, whether the sampling strategy is described and appropriate, the researcher(s) has identified potential sources of bias from their own position, ethics, the approach to analysis and its robustness, whether evidence supports any recommendations, and an overall score.

### 3.6 Data extraction

Covidence will be used as the data screening software, and Excel Spreadsheets will be used for data extraction. The shortlist of papers will be extracted from using standardised pro‐forma to ensure consistency of data extraction. We will pilot extraction with the pro‐forma as well as holding team meetings to build consensus about what to extract and how, in particular related to the qualitative (thematic) data where extraction is more subjective than for quantitative data.

For included studies, data will be extracted by a single reviewer (with a peer reviewer process to check accuracy) into an online form (enabling multiple simultaneous users) developed for this review. This form has been drafted (with its current structure provided as a supporting document below) with multiple individuals inputting into its design, and it will be tested via a dry run with an individual that has not yet seen it to examine its usability and ability to capture all the necessary information for the synthesis of findings. Once the study has passed the full‐text review, one reviewer will extract the necessary information into the extraction form, and another reviewer with a significant quantitative background will check the accuracy and relevance of the extracted information, in particular the extraction of quantitative results. Points of contention around the extraction, including when extracting datapoints that are subjective, for example, disadvantage/at risk characteristics, will be discussed with the wider review team through the Microsoft Teams channel dedicated to the research project before reaching a consensus verdict.

### 3.7 Synthesis

We will synthesise the literature by performing two forms of analysis. Firstly, we will aim for a statistical meta‐analysis where appropriate. Secondly, we will draw on process and implementation data in narrative form within thematic analysis.

#### 3.7.1 Meta‐analytic synthesis

##### Calculating effect sizes for meta‐analysis

To perform the meta‐analysis, we require standardised effect sizes. Where studies do not report these (as is common they may only report a treatment effect estimate along with some measure of dispersion), we will use the Campbell Collaboration's effect size calculator. If insufficient information is provided to calculate an effect size, we will contact the listed author to request additional information. Where additional information is acquired and used to calculate effect sizes, this additional information will be retained to be provided alongside the technical report should it be requested. For trials reporting outcomes only for participants completing the trial, the primary author will be asked to provide additional information to permit intention‐to‐treat analyses. Studies in which participants are analysed as members of the groups to which they were originally assigned (intention‐to‐treat analysis), studies that include only those participants who were willing or able to provide data (available‐case analysis), and studies that analyse participants who adhered to the study's design (per‐protocol analysis) will be analysed separately, sample size permitting – should this not be possible, we will transform impact estimates into a common treatment effect type using differences in participation rates between the treatment and control/comparison group. Where obtaining missing data is not possible or investigators are unresponsive, we will make assumptions regarding whether the data are ‘missing at random’ or ‘not missing at random’ and will follow the recommendations of the Cochrane Handbook for Systematic Reviews of Intervention. We will conduct sensitivity analysis around our assumptions to understand how they may affect our overall findings. Where studies have missing summary data, such as missing standard deviations, we will derive these where possible, using formulas provided in the Cochrane Handbook for Systematic Reviews of Interventions. We will specify the methods used to address any missing data in the results tables. If imputation was not possible, we will outline the reasons for this in the text.

For outcomes that are continuous variables, such as test scores, and reported on the same scale of measurement, we plan to use the mean difference (i.e., weighted mean difference). For outcomes reported on different scales, we plan to use Hedges’ *g* to report standardised mean differences (SMDs). We will report the 95% confidence intervals for mean differences and standardised mean differences. For dichotomous outcomes, such as whether in employment or not, where possible we will use (log) odds ratios. However, where this is not suitable (often the case with quasi‐experimental designs where directly comparing the likelihood of outcomes between the treatment and control groups does not account for endogeneity issues which the QED approach is looking to avoid), we will use the approach used for continuous outcomes. This is akin to the approach employed by Card et al. (2010) who faced this challenge when performing a meta‐analysis of active labour market policies. Should this not be possible due to missing information, we will pursue alternative options for constructing effect sizes, for instance by calculating Cohen's *d* instead of Hedges’ *g*.

If outcome measures are reported across studies using both binary and continuous data, two authors of this review will assess and discuss whether it is logical and appropriate in the context of the study and wider field to convert using lnOR = *g* × π/3^0.5. Time of outcome measurement will be recorded in months or years with endline = 0. We will consult experts from the Campbell Collaboration (Howard White) as necessary.

We anticipate that allocation to a particular matching intervention or process change will be on the individual level. In the event of clustering, for example on the community level, we anticipate that investigators will have controlled for a clustering effect in their approach. We will contact authors for further information if this is unclear. If the clustering effect was not controlled for, we will request individual participant data to calculate an estimate of the intra‐cluster correlation coefficient (ICC), and, if that is not available, we will obtain external estimates of the ICC from similar studies. We will analyse effect sizes and confidence intervals using appropriate software (such as Stata).

##### Meta‐analytic approach

Outcomes identified in the literature eligible for extraction will be categorised across the five outcome domains of interest. Provided sufficient sample sizes, meta‐analyses will be performed across each of these outcome domains. Within these domains, outcome measures will be grouped based upon the time period at which they are measured. If there are insufficient sample sizes to combine outcomes across multiple time period groupings, the data from the longest follow‐up that is based on the full sample (i.e., not affected by attrition) will be used. We will use the attrition guideline standards set by What Works Clearinghouse, accounting for different levels of overall and differential attrition as well as the primary investigator's judgement about whether the source of attrition is at random or endogenous. However, as previously mentioned, the initial literature review to scope existing evaluations of summer programmes identified that the outcomes measured were often relatively short term, with studies often not following‐up after programme end. As such, this may not be a significant issue faced by this review.

The main approach that we will use to estimate average effect size and variability will be the random effects model (REM)—a consensus approach commonly used in meta‐analysis. We will use random rather than fixed effects to enable the results of the analysis to be applicable beyond the included studies and given study heterogeneity (in terms of intervention population, form of the intervention, labour market context and so forth) it is unsound to assume that there is a common effect across the included interventions.

We will examine the heterogeneity in the extracted effect sizes, producing forest plots, as is standard practice. We will also statistically test for heterogeneity using the *I* ^2^ and Cochran's *Q*‐test, as recommended by the Cochrane Handbook for Systematic Reviews of Interventions (Deeks et al., 2022). Afterwards, we will estimate the average effect size across studies, vis‐à‐vis different outcomes, and the level of variability across those effect sizes. We also plan to perform various sub‐group analyses. The default approach we will use for this is meta‐regression analysis. This involves regressing the trial's effect size on characteristics of interest including those previously mentioned. The advantages of this approach include the ability to determine the sources of heterogeneity in effect sizes, and the ability to reveal and adjust for the confounding effects of the study, intervention and participant characteristics of interest (Stone et al., 2019). The characteristics of interest that will inform the previously mentioned sub‐group analyses will also inform the moderators that feature in the meta‐regression analysis. However, this approach requires a sufficient sample of studies to be performed. Therefore, our ability to perform a meta‐regression will depend on the resulting list of included studies, following screening. Statistical packages in Stata, such as *robumeta*, will be used to perform this analysis. Should meta‐regression not be possible, we will use the details about the study characteristics as well as characteristics of the participants and the intervention (see the data extraction form below) that we will extract to construct sub‐groups of studies. As well as comparing the impact of summer employment programmes versus summer educational programmes, other characteristics of interest that study sub‐group analysis may be performed on include whether the evaluated intervention is UK or non‐UK based, the form(s) of disadvantage or at risk characteristic(s) that participants exhibit, whether the intervention is in whole or in part (e.g., it is accompanied by after‐school activities) a summer programme, the types of activities that comprise the intervention, the intensity of the programme (hours a day, number of days and weeks, etc.), and whether there is an aspect of personalisation of the programme to the specific needs of the participant. Which sub‐group analyses are possible is also dependent on the sample sizes across sub‐groups and the quality of information provided by studies relating to the study, participant and intervention characteristics of interest. In performing sub‐group analyses, we will consider the impact of multiplicity and follow guidance from the Cochrane Handbook in addressing this issue.

When reporting our results, the average effect sizes found will be back‐transformed into a metric of relevance, so as to better place them in an understandable and policy‐relevant context. Several forms of sensitivity analysis will also be performed, such as the exclusion of low‐quality trials (in the knowledge that this may introduce collider bias in the context of the sensitivity analysis), and one‐way sensitivity analyses involving the sequential removal of studies to determine which ones drive the pooled results.

One issue that may be faced in this study's meta‐analytical component is effect size dependence. As we are interested in outcome measures that are highly interrelated and will likely be measured within the same study, we may end up including several correlated outcomes measured in the same sample. This will reduce the information provided by the effect size estimates taken from said studies. If multiple outcome measures are reported per specific outcome domain per study, we will select only the most relevant/most commonly used outcome measure to represent the specific interventions impact on that domain and/or consider producing estimates of average effect sizes across these specific outcome measures. Where necessary, we will combine results for separate niche outcomes or outcomes measured across multiple time points together in separate initial meta‐analysis, with the combined effect size estimates then entered into the main meta‐analysis for the final outcome of interest.

Whilst we will aim to conduct a meta‐analysis, depending on the specifics of the literature that the search process surfaces a meta‐analysis may not be feasible or appropriate. Potential reasons for this provided in the Cochrane handbook are: if the studies surfaced are too diverse, combining their results may be nonsensical as this would ‘combine apples with oranges’; if there is risk of bias amongst the studies to be included, meta‐analysis would compound these errors and produce incorrect results; if there is publication or reporting bias, meta‐analysis will produce an inappropriate summary of the true effect size of the intervention type. Should we determine that a meta‐analysis is not the appropriate approach to synthesise the quantitative data, we would follow the Synthesis Without Meta‐analysis (SWiM) guidelines developed by BMJ (2020), which details ten items to be covered in the alternative synthesis approach (Grouping studies for synthesis; Describe the standardised metric and transformation methods used; Describe the synthesis methods; Criteria used to prioritise results for summary and synthesis; Investigation of heterogeneity in reported effects; Certainty of evidence; Data presentation methods; Reporting results; Limitations of the synthesis).

#### 3.7.2 Approach to thematic analysis

Since this systematic review also seeks to identify components and features shared across and between successful summer programmes (and specific to job separately from education programmes), we will also attend to qualitative evaluations of the interventions examined in the included studies. This poses a methodological challenge: whilst, with quantitative studies, the data extraction process is relatively straightforward, there is greater variability in reporting and analytical methods in qualitative research, which in turn makes data extraction more difficult (Lucas et al., 2007).

We will pilot our approach through team workshops and pilot coding with review to ensure our understandings of contexts, causal mechanisms, and facilitators and barriers are shared, and fixed onto a plausible causal pathway. A codebook will then be developed to guide our extraction of studies. We will pilot coding and hold team reviews to ensure there is sufficiency in the detail coded to support later analysis. Using the codebook means that studies will be coded according to a predefined theoretical framework (Thomas & Harden, 2008). However, it is important to note that not all codes and themes can be predefined; it is inevitable for each coder to extract inductive themes in the process (Harden & Thomas, 2005). We will facilitate this by adding subcodes both in initial coding and then in later synthetic analysis to deepen understanding through a dedicated Microsoft Teams channel. While, thematic synthesis introduces a significant level of subjectivity into the analysis, this will be reduced by making the process more transparent by including information in the codebook on whether each code was predefined or defined inductively, as recommended by Fereday and Muir‐Cochrane (2006).

The thematic synthesis for this systematic review will proceed according to the steps below, as outlined by Thomas and Harden (2008):

1. Line‐by‐line coding
2. Organising codes along a hierarchical coding structure
3. Interpreting analytical themes.

To achieve the final step will involve interrogating the Excel workbook codes on each main theme and the related subthemes, drawing and testing patterns within each to arrive at a synthesis of key issues as well as factors that outline these. Team workshops throughout will enable discussion and consensus building to ensure consistency in approach. Two members of the team will collaborate to interrogate the data against each theme, which will act against bias emerging as data is broken down by programme, population, and outcome type. The Excel framework and coding will provide the underpinning evidence for the decisions reached.

We will follow a thematic synthesis approach which combines elements of meta‐ethnography and grounded theory through inductive development and ‘constant comparison’. Thematic synthesis allows for different methodologies, contexts, and subject focuses to be combined and so will be appropriate for this review. It is also useful for generating new hypotheses which will add value particularly as we explore the comparative effects of summer education versus summer employment programmes.

The thematic analysis will also serve the key purpose of enabling us to capture information related to the causal pathway, assumptions, moderators and contexts, as well as subgroups. Some of the themes that we assume will emerge from the review, could for example include the role of targeting and marketing to support positive engagement, engagement with new or different environments, people, and experiences, and the role played by relationships built with staff in building confidence to transition to and succeed in positive destinations. This process will also allow us to identify implementation issues and insights into how results could be replicated in practice, including what the critical success factors are for delivery.

### 3.8 Reporting

We will report our findings in a systematic review paper. The template and structure for the paper will be agreed with YEF, YFF and the Campbell Collaboration before reporting commences. An initial draft of the paper will be submitted to YEF, YFF and the Campbell Collaboration for feedback and comment, after which an edited and finalised systematic review paper will be submitted.

In reporting the findings of the meta‐analysis and the thematic synthesis, we will abide by the requirements laid out by the Methodological Expectations of Campbell Collaboration Intervention Reviews’ (MECCIR's) reporting standards (The Methods Group of the Campbell Collaboration, 2019). The findings of the thematic analysis will be reported in a narrative synthesis.

The target audience of the systematic review paper includes the users of the YEF and YFF toolkits—policy makers (including the Department for Education, Department for Work and Pensions and the Ministry of Justice), the ‘what works’ movement focused on children and youth support, youth support organisations (such as Youth Employment UK, Princes Trust and National Youth Agency), and providers of summer programmes. The report will sit alongside other technical reports for the YEF and YFF toolkits.

Becci Newton will take overall responsibility for the drafting of the paper, with ultimate control of content decisions. Daniel Muir will lead the contribution on technical synthesis of the meta‐analysis. Becci Newton will lead on drafting the narrative synthesis of the thematic analysis with close support from Cristiana Orlando. Other member of the wider review team will contribute to both the technical synthesis of the meta‐analysis and narrative synthesis of the thematic analysis.

## CONTRIBUTIONS OF AUTHORS

IES brings capabilities in the areas that matter most for this project—methodological expertise in reviews; deep experience in the labour market, skills, and training; and expertise in working collaboratively with other organisations to achieve shared goals and produce resources that enable evidence to be put into practice.

- **Content and qualitative synthesis:**
  ∘ **Becci Newton** has over 20 years’ experience of applied social research and evaluation and is a recognised expert on topics including: young people's transitions, particularly within the 14–19 phase; further education and Apprenticeship; and unemployment, inactivity and welfare‐to‐work including vulnerable and disadvantaged young people including those who are NEET. Cross cutting themes she covers include equality and diversity, social justice, and overcoming poverty and disadvantage. She is co‐PI for the YFF Employment Toolkit semi‐systematic reviews with meta‐analysis, alongside Ellie Ott at CEI. As one of the project's co‐principal investigators, Becci provides content expertise to guide the evidence search and analysis process. In addition, she will supervise the reporting process, ensuring that research outputs are of an excellent standard and are usable to policy audiences.
  ∘ **Cristiana Orlando** brings expertise on youth employment and the experience of young pepe who are at risk. She is leading a large‐scale action oriented research for the Health Foundation into improving the quality of work young people access. Cristiana was also involved in the data extraction and thematic analysis of one of the rapid evidence assessment for the YFF youth employment toolkit.
- **Systematic review methods, information retrieval and statistical analysis**:
  ∘ **Daniel Muir** has worked on a variety of projects, including the YFF's youth employment toolkit. Commissioned by the YFF, and working alongside the Centre for Evidence and Implementation, the development of the youth employment toolkit, which seeks to improve understanding of the barriers young people face when entering the labour market, has involved semi‐systematic rapid evidence assessments in the field of youth employment and careers. Daniel lead the meta‐analysis (both conducting the analysis and the reporting of) for one of the rapid evidence assessments, and has been heavily involved in the data extraction of the other. Given his experience with evidence assessments, he will help retrieve and screen articles obtained from the electronic database search, extract data from identified literature, perform the study quality assessment, and conduct of the meta‐analysis. He will be supported in applying the systematic review methods and information retrieval by Alexandra Nancarrow who managed one of the aforementioned rapid evidence assessments for the development of YFF's youth employment toolkit. Alexandra has a PhD in developmental psychology and expertise in quantitative analysis.

Other members of the IES staff will also be enlisted to provide further support to this review. This includes our Principal Impact Economist who will support data extraction and meta‐analysis, and research officers who have contributed to the recent YFF toolkit reviews and bring experience of information retrieval, data extraction for quantitative and qualitative studies.

## DECLARATIONS OF INTEREST

The authors declare no potential conflicts of interest.

**Schedule**

We estimate that the major steps of the systematic review will occur as follows:

1. Protocol finalised: January 2022
2. Search and screening: January 2022–February 2023
3. Data extraction and analysis: February–April 2023
4. Draft report: April 2023
5. Final report: May 2023

## SOURCES OF SUPPORT

**Internal sources**

- No sources of support provided

**External sources**

- This review is being co‐financed by the Youth Endowment Fund and the Youth Futures Foundation.
